# Impact of Nitric Oxide Bioavailability on the Progressive Cerebral and Peripheral Circulatory Impairments During Aging and Alzheimer's Disease

**DOI:** 10.3389/fphys.2018.00169

**Published:** 2018-03-14

**Authors:** Massimo Venturelli, Anna Pedrinolla, Ilaria Boscolo Galazzo, Cristina Fonte, Nicola Smania, Stefano Tamburin, Ettore Muti, Lucia Crispoltoni, Annamaria Stabile, Alessandra Pistilli, Mario Rende, Francesca B. Pizzini, Federico Schena

**Affiliations:** ^1^Department of Neurosciences, Biomedicine and Movement Sciences, University of Verona, Verona, Italy; ^2^Department of Medicine, University of Verona, Verona, Italy; ^3^Department of Computer Science, University of Verona, Verona, Italy; ^4^Neuromotor and Cognitive Rehabilitation Research Centre, University of Verona, Verona, Italy; ^5^Mons. Mazzali Foundation, Mantua, Italy; ^6^Department of Surgical and Biomedical Sciences, Section of Human Anatomy, School of Medicine, University of Perugia, Perugia, Italy; ^7^Neuroradiology, Department of Diagnostics and Pathology, Verona University Hospital, Verona, Italy

**Keywords:** circulation, aging, Alzheimer's disease, nitric oxide, vascular dysfunction

## Abstract

Advanced aging, vascular dysfunction, and nitric oxide (NO) bioavailability are recognized risk factors for Alzheimer's disease (AD). However, the contribution of AD, *per se*, to this putative pathophysiological mechanism is still unclear. To better answer this point, we quantified cortical perfusion with arterial spin labeling (PVC-CBF), measured ultrasound internal carotid (ICA), and femoral (FA) artery blood flow in a group of patients with similar age (~78 years) but different cognitive impairment (i.e., mild cognitive impairment MCI, mild AD-AD1, moderate AD-AD2, and severe AD-AD3) and compared them to young and healthy old (aged-matched) controls. NO-metabolites and passive leg-movement (PLM) induced hyperemia were used to assess systemic vascular function. Ninety-eight individuals were recruited for this study. PVC-CBF, ICA, and FA blood flow were markedly (range of 9–17%) and significantly (all *p* < 0.05) reduced across the spectrum from YG to OLD, MCI, AD1, AD2, AD3 subjects. Similarly, plasma level of nitrates and the values of PLM were significantly reduced (range of 8–26%; *p* < 0.05) among the six groups. Significant correlations were retrieved between plasma nitrates, PLM and PVC-CBF, CA, and FA blood flow. This integrative and comprehensive approach to vascular changes in aging and AD showed progressive changes in NO bioavailability and cortical, extracranial, and peripheral circulation in patients with AD and suggested that they are directly associated with AD and not to aging. Moreover, these results suggest that AD-related impairments of circulation are progressive and not confined to the brain. The link between cardiovascular and the central nervous systems degenerative processes in patients at different severity of AD is likely related to the depletion of NO.

## Introduction

Alzheimer's disease (AD) is the most common form of dementia, with an attested prevalence of ~24 million which is predicted to quadruplicate by 2050 (Reitz et al., 2011). Pathophysiological mechanisms of AD are well-defined, including diffuse neuritic extracellular amyloid (Aβ) plaques and intracellular neurofibrillary tangles coupled with reactive microgliosis, loss of neurons and synapses in the cortex (Reitz and Mayeux, 2014). From the vascular point of view, Aβ peptide accumulation in the tunica media and adventitia of cerebral blood vessels, a condition termed cerebral amyloid angiopathy (CAA), is associated with vessel smooth muscle cell degeneration, resulting in impaired cerebral circulation (Maier et al., 2014).

Apart from this direct effect of Aβ accumulation in the cortical neurons and vessels, there is increasing evidence that AD is associated with several dysregulated processes, which affect brain and systemic circulation, suggesting that vascular dysfunction may play a role in the pathogenesis of AD (Iturria-Medina et al., 2016). These pieces of evidence pose the question whether AD is an age-related neurodegenerative disorder with vascular consequences, or a vascular disorder with neurodegenerative sequels (De La Torre, 2010). Indeed, both aging and AD appear to be involved in the decline of the systemic and cerebral vascular function (De La Torre, 2009). However, the contribution of AD, *per se*, to vascular changes is still unclear. Additionally, the cardiovascular and the central nervous system (CNS) changes have been postulated to occur in parallel during the progression of AD (Picano et al., 2014).

In the brain of patients with AD, diffuse cortical changes have been demonstrated (Dallaire-Theroux et al., 2017), coupled with a reduced perfusion of the temporo-parietal association cortices, mesial temporal structures and the frontal association cortex (Herholz, 2011). However, potential reduction of blood flow availability inward from extracranial conduit arteries (i.e., internal carotid artery (ICA), vertebral artery) may also contribute to the onset of AD (Liu et al., 2014; Clark et al., 2017). In this scenario, nitric oxide (NO) is considered the most important vasodilator factor responsible for the preservation of vasomotor function (Katusic and Austin, 2014). Indeed, reduced availability of NO in both cerebral and peripheral vessels results in major detrimental alterations of vascular function (Katusic and Austin, 2014). However, the role of NO bioavailability in the control of extracranial blood flow, cerebral, and systemic circulation in patients with different AD severity have not been so far fully elucidated.

Therefore, the aims of the present study were two-fold. The first was to evaluate if the NO bioavailability, cerebral perfusion, extracranial, peripheral blood flow and systemic vascular function are reduced in AD in comparison to healthy young and old individuals. The second was to compare these measures in patients with different AD severity. Specifically, we have assessed cortical perfusion with arterial spin labeling (ASL) Magnetic Resonance Imaging (MRI), and measured ICA and femoral (FA) artery blood flow in young (YG) and old (OLD) healthy controls, patients with mild cognitive impairment (MCI) and AD of different severity: mild AD (AD1), moderate AD (AD2), and severe AD (AD3). NO bioavailability was determined in the six groups via plasma NO metabolites (nitrite and nitrate). Passive leg-movement (PLM) induced hyperemia was used to assess both endothelial NO availability and systemic vascular function. We hypothesized that (a) the severity of AD would impact on cortical perfusion, as well as ICA and FA blood flow, and (b) brain and systemic impairment of circulation would be associated with a depletion of NO bioavailability.

## Methods

### Participants

Patients with MCI and AD were recruited from the Neuromotor and Cognitive Rehabilitation Research Center Azienda Ospedaliera Universitaria Integrata of Verona, and the Geriatric Institute Mons. Arrigo Mazzali Foundation (Mantua, Italy). Clinical diagnosis of MCI and probable AD was established according to the National Institute on Aging-Alzheimer's Association diagnostic guideline for MCI due to AD and AD (Albert et al., 2011; Mckhann et al., 2011). All patients had a previous neuroimaging study (MRI or CT) to support the diagnosis of MCI and/or probable AD.

Dementia severity was assessed by means of the Mini Mental State Examination (MMSE) (Folstein et al., 1975) and the Clinical Dementia Rating scale (CDR) (Morris, 1993). According to the severity of dementia, patients with AD were divided in three groups: AD1 with MMSE scores between 20 and 24 and CDR 1, AD2 with MMSE scores between 10 and 19 and CDR 2, and AD3 with MMSE scores lower than 10 and CDR 3. Two additional healthy control groups (i.e., YG and OLD) were recruited from the same geographical area, after a physician's assessment of negligible cardiovascular and musculoskeletal diseases. This screening included health history, physical examination, blood pressure assessment, blood sample, and familiarization with the study procedures. OLD had to have a MMSE ≥ 24. As reported in the AD diagnostic guidelines (Mckhann et al., 2011), individuals with a diagnosis of vascular dementia (VaD) were not included in the study. Other exclusion criteria were: history of depression or psychosis, alcohol or drug abuse, other neurological (e.g., Parkinson's disease, traumatic brain injury, stroke, multiple sclerosis), cardiac, orthopedic (e.g., osteoarthrosis) or respiratory conditions (e.g., chronic obstructive pulmonary disease). All experiments were conducted after informed and written consent was obtained from the patients and their relatives and healthy individuals in accordance with the Declaration of Helsinki, as part of a protocol approved by the Institutional Review Board of the Azienda Ospedaliera Universitaria Integrata (Approval #2389).

### Assessment procedure

Neurologists and clinical neuropsychologists with a specific expertise in dementia investigated the cognitive profile of the patients with a full neuropsychological profile and the following tests were performed. The MMSE (Folstein et al., 1975) was used to assess the global cognitive status. The CDR (Morris, 1993) scale was administered to quantify the severity of dementia. The Italian version of the Frontal Assessment Battery (FAB) (Appollonio et al., 2005) was used to assess executive functions.

### Level of physical activity

The International Physical Activity Questionnaire (IPAQ) (Booth, 2000) was used to estimate the level of physical activity of the participants. Each question was administered to the healthy volunteers and to the patient's caregivers.

### Volume anthropometry assessment

Thigh and lower leg volume were calculated based on leg circumferences (three sites: distal, middle, and proximal), thigh and lower leg length, and skinfold measurements using the following formula:

$$V=\;\frac{L}{12\pi \;}\;\cdot \left(C1^{2}+C2^{2}+C3^{2}\right)-\frac{S-0.4}{2}\;\cdot L\cdot \;\frac{C1+C2+C3}{3}$$

where L refers to the length; C1, C2, and C3 refer to the proximal, middle, and distal circumferences, respectively; and S is skinfold thickness of either the thigh or the lower leg. The length of the leg was measured from the greater trochanter to the lateral femoral epicondyle (thigh) and from the head of the fibula to the lateral malleolus (lower leg). The length and circumference were measured to the nearest 1 mm using a flexible standard measuring tape. Skinfold thickness was measured using skinfold calipers (Beta Technology Incorporated, Cambridge, MD) at three sites at the midpoint of each limb segment (Layec et al., 2014).

### Resting oxygen uptake assessment

Briefly, oxygen uptake was recorded with the subjects supine and at rest for 20 min. Expired gases were analyzed on a breath-by-breath basis by a metabolimeter (K4 b^2^, Cosmed, Rome, Italy).

### Cortical perfusion assessment

A subgroup of the total population underwent MRI to assess non-invasively cerebral blood flow (CBF) with ASL (Detre et al., 1992). In details, forty-three subjects (YG: 10, OLD: 7, MCI: 6, AD1: 5, AD2: 9, AD3: 6) were scanned on a 3T Philips Achieva system equipped with an 8-channel head coil. They were instructed to lie as still as possible in the scanner, to keep their eyes closed but not to fall asleep while images were collected. For ASL data, pseudo-continuous (pCASL) labeling was acquired using the following parameters: TR/TE = 4,400/11 ms; label duration/post-label delay = 1,650/1,800 ms; 45 Control/Label volumes; 26 slices, 3 × 3 × 3 mm^3^, slice gap = 1 mm; two background suppression pulses at 1,700 and 2,926 ms from the start of the scan. A calibration scan with the same parameters as the ASL sequence but longer TR (10 s) and no background suppression was also acquired to estimate the equilibrium magnetization. Finally, a 3D T1-weighted turbo field echo anatomical scan was also acquired for each subject (TR/TE = 8.16/3.73 ms; 180 slices, 1 × 1 × 1 mm^3^).

ASL data were preprocessed and analyzed using FSL 5.0.9 (FMRIB, Oxford, UK) and Matlab 7.14 (MathWorks, Natick, MA). ASL data were first corrected for nuisance effects (head motion profiles, cerebrospinal fluid (CSF) and white matter (WM) signals) by using linear regression that minimizes the sum of squares of the residuals. The ASL calibration scan was used for estimating the coregistration parameters from ASL to the individual T1-weighted image by applying a 3D rigid-body registration with a normalized mutual-information cost function and 7 degrees of freedom.

Pre-processed Control and Label volumes were then surround subtracted and averaged to obtain perfusion-weighted images. These perfusion-weighted maps were quantified into CBF [ml/100 g/min] applying the general kinetic model (Buxton et al., 1998) as follows:

$$CBF=\;\frac{6000\cdot \lambda \cdot \Delta M\cdot e^{\frac{PLD}{T_{1b}}}}{2\cdot \alpha \cdot \alpha_{inv}\cdot T_{1b}\cdot M_{0t}\cdot (1-e^{-\frac{\tau }{T_{1b}}})}$$

where λ is the brain-blood partition coefficient (0.9 mL/g), ΔM represents the difference images (perfusion-weighted maps), PLD is the post-labeling delay, T_1b_ is the longitudinal relaxation time of arterial blood (1,650 ms), α is the labeling efficiency (0.85 for pCASL), where α_inv_ corrects for the decrease in labeling efficiency due to two background suppression pulses (0.83). M_0t_ is the tissue equilibrium magnetization, voxel-wise estimated from the calibration scan, and τ represents the labeling duration (Alsop et al., 2015). The increase in label decay in the ascending slices acquired with 2D readout was accounted for.

In order to perform partial volume effects-correction (PVC) mainly related to the low ASL spatial resolution and brain atrophy, partial volume-corrected cortical and WM flow maps were created for each subject. In details, high resolution gray matter (GM) and WM probability maps from the segmentation of the 3D T1-weighted image were first smoothed with a 3 × 3 × 3 mm^3^ kernel to mimic the ASL resolution. These smoothed maps were then down sampled to the ASL space using the inverse of the previously estimated transformation matrix and finally applied to the CBF maps for PVC following the equation I_corr_ = I_uncorr_/(P_gm_ + 0.4·P_wm_) (Du et al., 2006). For each subject, the mean corrected CBF value within the GM mask was calculated and used as individual representative measure of the whole cortical perfusion.

To provide group CBF maps, individual T1-weighted images were registered to the Montreal Neurological Institute (MNI) space with 1 × 1 × 1 mm^3^ resolution using a non-linear method (FNIRT tool in FSL) and the joint ASL/T1-weighted and T1-weighted/MNI space transformation parameters were used to spatially normalize the subject specific CBF maps in this common space. Representative mean uncorrected (whole brain) and PVC cortical (GM only) CBF maps in MNI space were finally derived for each of the six groups.

### ICA and FA blood flow assessment with doppler ultrasound imaging

ICA, and FA artery diameters and blood velocities were recorded with the subjects supine and at rest for 20 min. When the blood flow was stable, 1-min video of the above-mentioned arteries was recorded on the ultrasound system. Specifically, the Doppler probe was positioned at the level of ICA ~1 cm above the common carotid bifurcation, and at the level of the common FA, distal to the inguinal ligament and proximal to the deep and superficial femoral bifurcation. Triplex Doppler clips were recorded with a Logiq-7 ultrasound Doppler system (General Electric Medical Systems, Milwaukee, WI, USA). The ultrasound Doppler system was equipped with a 12-14 MHz linear array transducer. Artery diameter was determined at a 90° angle along the central axis of the scanned area. Blood velocity (V_mean_) was measured using the same probe utilizing a frequency of 5 MHz. Measurements of V_mean_ were obtained with the probe positioned to maintain an insonation angle of 60° or less and the sample volume was centered and maximized according to vessel size. Utilizing arterial diameter and V_mean_, blood flow was calculated as:

$$\text{Blood flow}={\text{V}}_{\text{mean}}\;\cdot \pi \;\cdot \;{\left(\text{vessel diameter}/2\right)}^{2}\;\cdot \;60$$

where blood flow is in milliliters per minute. To perform muscle and brain volume effect-corrections, mostly related to the lower limb muscle and brain atrophy, FA and ICA blood flow were normalized to leg muscle volume (thigh + lower leg volume) and total brain tissue volume (cortical, subcortical GM and WM volumes, including the brainstem and cerebellum), respectively (Liu et al., 2014; Venturelli et al., 2014). All scanning and blinded analyses were performed by experienced and skilled sonographers.

### NO bioavailability via plasma nitrates assessments

It is important to note, that all the participants were asked to refrain from oral intake of supplements or nutrients with elevated levels of nitrates. Specifically, on the 3 days before the assessments strawberries, lettuce, beets, and carrots were not included in the participants diet. Venous peripheral blood (25 mL) was collected between 9:00 and 10:00 am from patients and healthy controls in a fasted state and processed within 2 h to obtain measurements of blood glucose, number of red blood cells (RBC), hemoglobin (Hb), high- and low-density lipoprotein (HDL, LDL). From a different vacutainer, plasma was separated from peripheral blood by centrifugation (1,200 rpm for 20 min at 4°C) and kept at −80°C until analysis. Plasma samples were ultrafiltrated through a 30 kDa molecular weight cut-off filter (cat. No UFC503096) (Millipore, Molseheim, France) to reduce background absorbance. Nitrate concentration was detected by nitrate/nitrite colorimetric assay kit (cat. No 780001) (Cayman Chemical Co, Ann Arbor, MI, USA) according to the manufacturer's protocol. The detection limit of nitrate was 2.5 μM. The nitrate concentration was analyzed in duplicate and read against the manufacturer standard curve.

### Endothelial NO bioavailability and systemic vascular function via PLM

Recent investigations have revealed that PLM-induced hyperemia is predominantly a consequence of NO mediated vasodilation (Trinity et al., 2012). Therefore, we have adopted this noninvasive and reliable method to determine endothelial NO bioavailability. Moreover, the PLM protocol has been successfully adopted to determine systemic vascular function in healthy young (Mcdaniel et al., 2010a), elderly (Mcdaniel et al., 2010b), patients with spinal cord injury (Venturelli et al., 2014), and heart failure (Ives et al., 2016). During this evaluation, the subjects rested in the upright-seated position for 20 min before the start of data collection and remained in this position throughout this part of the study. The PLM protocol consisted of 60 s of resting baseline femoral blood flow data collection, followed by 60 s of passive knee extension and flexion with the same measure. PLM was performed by a member of the research team, who moved the subject's lower leg through a 90° range of motion (180-90° knee joint angle) at 1 Hz. Blood V_mean_ was analyzed with 1 Hz resolution on the Doppler ultrasound system (GE Logiq-7) for 60 s at rest and second by second for the first 60 s following the initiation of PLM. Relative changes (Δpeak) from rest of femoral blood flow was determined for each subject. To perform muscle volume effect-correction related to the skeletal muscle atrophy, Δpeak blood flow was normalized by thigh muscle volume (Venturelli et al., 2014).

### Data analysis and interpretation

The representative outcomes from each assessment were analyzed using a statistical software package (StatPlus:mac, AnalystSoft Inc.,-statistical analysis program for Mac OS®. Version v6.). The normal distribution of the sampling was checked by the Shapiro-Wilk test. A one-way analysis of variance (ANOVA), and, where indicated, a Tukey *post hoc* test, were used to determine the group differences. A chi-square (χ^2^) analysis was used to establish differences between categorical variables. Pearson correlation test was used to examine the correlation between variables. Significance was set at an α level of 0.05 (two-tailed), and the results are presented as mean ± SE.

## Results

### Characteristics of the participants

Demographic and clinical characteristics of the study participants are displayed in Table 1. Ninety-eight individuals (YG:10, OLD:14, MCI:19, AD:55) were recruited for this study. Except for the YG, all groups were matched for age, sex, body mass, thigh muscle volume, lower leg muscle volume, coexisting chronic conditions. Drugs for AD and other medications taken by the four groups of MCI/AD patients and the two groups of healthy individuals are displayed in Table 1.

**Table 1:** Demographic and clinical characteristics of the study participants.

	**YG (*N* = 10)**	**OLD (*N* = 14)**	**MCI (*N* = 19)**	**AD1 (*N* = 24)**	**AD2 (*N* = 20)**	**AD3 (*N* = 11)**
Sex	4♂-6♀	6♂-6♀	9♂-10♀	9♂-15♀	6♂-14♀	2♂-9♀
Age (years)	28 ± 2	76 ± 6^*^	77 ± 4^*^	78 ± 7^*^	80 ± 8^*^	80 ± 7^*^
Weight (kg)	68 ± 20	73 ± 12	75 ± 19	63 ± 12	73 ± 13	62 ± 4^§^
Height (m)	1.69 ± 0.3	1.67 ± 0.1	1.65 ± 0.1	1.58 ± 0.1	1.62 ± 0.1	1.62 ± 0.2
Lower limb volume (l)	8.3 ± 1.7	8.3 ± 1.5	7.8 ± 1.6	7.4 ± 1.4	7.4 ± 1.1	7.3 ± 1.3
Thigh volume (l)	5.9 ± 1.5	6.2 ± 1.3	5.7 ± 1.4	5.4 ± 0.9	5.1 ± 2.0	5.3 ± 1.3
Leg volume (l)	2.4 ± 0.7	2.1 ± 0.6	2.1 ± 0.9	2.0 ± 0.5	2.3 ± 1.1	2.0 ± 0.7
SBP (mm Hg)	118 ± 20	129 ± 33	136 ± 37	132 ± 12^*^	130 ± 9^*^	125 ± 9^*^
DBP (mm Hg)	85 ± 10	90 ± 24	92 ± 12	86 ± 10	90 ± 5	82± 10
Glucose (mg·dl^−1^)	88 ± 12	95 ± 32	107 ± 21^*^	91 ± 8^§^	95 ± 47^§^	89 ± 15^§^
RBC (10^6^·μl^−1^)	5.2 ± 0.9	5.0 ± 0.6	4.8 ± 0.4	4.8 ± 0.6	4.5 ± 0.5^*^^†^^§^	4.3 ± 0.3^*^^†^^§^
Hb (g·dl^−1^)	15 ± 2	15 ± 3	13 ± 1^*^^†^	13 ± 2^*^^†^	13 ± 2^*^^†^	12 ± 0.6^*^^†^
HDL (mg·dl^−1^)	49 ± 24	50 ± 17	58 ± 19	57 ± 21	57 ± 11	64± 17^*^^†^^‡^^¶^
LDL (mg·dl^−1^)	99 ± 30	100 ± 23	102 ± 15^*^	122 ± 32^*^^§^	110 ± 12^*^^‡^	136 ± 18^*^^§^
Education (years)	19 ± 2	10 ± 6^*^	10 ± 4^*^	9 ± 5^*^	7 ± 4^*^	7 ± 4^*^
IPAQ (METs·min·week^−1^)	12,340 ± 832	4,043 ± 548^*^	3,874 ± 655^*^	4,129 ± 438^*^	3,784 ± 732^*^	3,833 ± 543^*^
Resting oxygen uptake (ml·m^−1^·kg^−1^)	4.4 ± 1.8	3.9 ± 1.8	3.6 ± 1.1	4.5 ± 1.2	3.5 ± 1.8	3.7 ± 1.5
ExpCO_2_ (ml)	27.5 ± 7.8	23.2 ± 9.2	17.8 ± 9.8	21.9 ± 14.3	15.0 ± 5.5	19.5 ± 7.8
**CLINICAL CHARACTERISTICS**						
Time since diagnose of MCI or AD (years)	–	–	2 ± 1	6 ± 2 ^§^	8 ± 3^§^^‡^	8 ± 2^§^^‡^
MMSE (0-30)	–	28 ± 1	27 ± 2^†^	22 ± 3^†^^§^	16 ± 3^†^^§^^‡^	11 ± 4^*^^†^^§^^‡^^¶^
CDR (0-3)	–	–	0.5	1^§^	2^§^^‡^	3^§^^‡^^¶^
FAB (0-18)	–	–	12 ± 2	9 ± 3^§^	8 ± 3^§^	4 ± 4^§^^‡^^¶^
**PHARMACOLOGICAL TREATMENT n. (%)**						
Cholinesterase Inhibitors	0	0	2 (10)	10 (42)^*^^†^	5 (25)^*^^†^	3 (27)^*^^†^
Antipsychotics	0	0	0	1 (4)	1 (5)	1 (9)
Antidepressants	0	0	0	2 (8)	4 (20)^*^^†^	2 (18)^*^^†^
Benzodiazepines	0	0	0	0	1 (5)	1 (5)
**COMORBIDITY n. (%)**						
Cardiovascular diseases	0	0	1 (5)	4 (16)^*^^†^	2 (10)^*^^†^	3 (27)^*^^†^
Diabetes	0	0	1 (5)	0	1 (5)	1 (9)
Arthrosis	0	0	1 (5)	2 (8)	1 (5)	1 (9)

*♂, male; ♀, female; MCI, Mild Cognitive Impairment; AD, Alzheimer's Disease; SBP, systolic blood pressure; DBP, diastolic blood pressure; RBC, red blood cells; Hb, hemoglobin; HDL, high-density lipoprotein; LDL low-density lipoprotein; IPAQ, international physical activity questionnaire; ExpCO_2_, expired carbon dioxide; MET, metabolic equivalent; MMSE, Mini Mental State Examination; CDR, Clinical Dementia Rating Scale; FAB, Frontal Assessment Battery. Values are expressed as mean ^±^standard deviation (or percentage in brackets)*.

* p < 0.05 vs. YG;

† p < 0.05 vs. OLD;

§ p < 0.05 vs. MCI;

‡ p < 0.05 vs. AD1;

¶ *p < 0.05 vs. AD2*.

### Resting metabolism, and level of physical activity

Resting oxygen uptake, expired carbon dioxide (ExpCO_2_) and the values of IPAQ, taken as marker of basal metabolism and level of physical activity, respectively, are illustrated in Table 1. Interestingly, any statistical difference was retrieved among the six groups in terms of basal metabolism (all *p* > 0.2). In comparison to the YG, the level of physical activity was significantly reduced in OLD (*p* < 0.01), MCI (*p* < 0.01), AD1 (*p* < 0.01), AD2 (*p* < 0.01), and AD3 (*p* < 0.01). However, the differences in IPAQ values among OLD, MCI, AD1, AD2, and AD3 were not significant and negligible.

### Cortical perfusion

**Figure 1:**
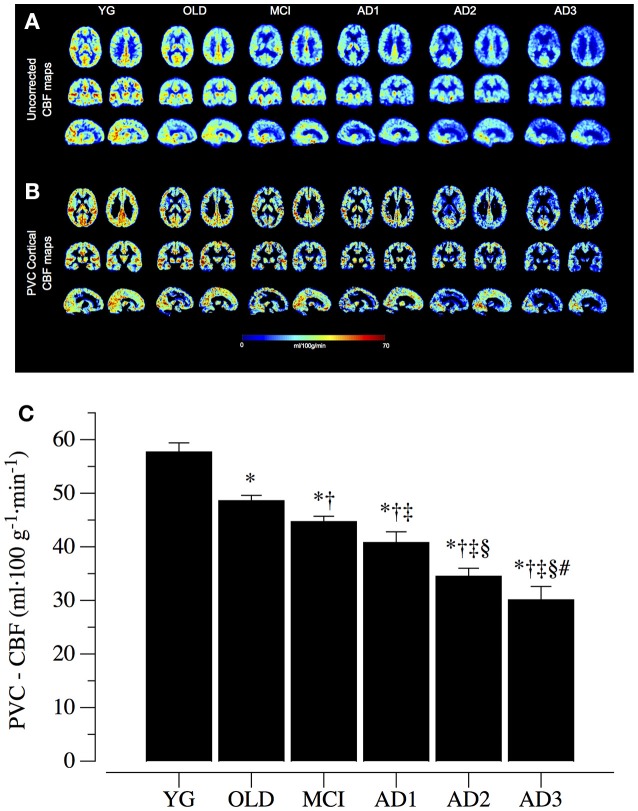
Cortical perfusion data. **(A)** Group whole brain uncorrected cerebral blood flow (CBF) maps, calculated as average across the individual subjects belonging to healthy young (YG) and healthy old (OLD) controls, patients with mild cognitive impairment (MCI) and with different severity of AD, namely mild AD (AD1), moderate AD (AD2), and severe AD (AD3). Two representative sections are reported for each of the three views (axial, coronal, and sagittal). **(B)** Group mean cortical CBF maps after partial volume correction (PVC cortical), reported for the same anatomical levels as before. **(C)** reports group mean PVC cortical CBF levels (mean ± S.E). ^*^Significantly different vs. YG group; ^†^Significantly different vs. OLD group. ^‡^Significantly different vs. MCI group. ^§^Significantly different vs. AD1 group. ^#^Significantly different vs. AD2 group.

Representative mean CBF images for the six groups are shown in Figure 1, reporting both whole brain uncorrected (Figure 1A) and PVC cortical (Figure 1B) CBF maps at the same anatomical level. As clearly visible, there was a marked and progressive reduction in the CBF parameter, confirmed after PVC, across the spectrum from YG to OLD, MCI, AD1, AD2, and AD3 subjects. Quantitative analysis supported these visual impressions (*p* < 0.05 among the six groups) with average PVC-CBF levels of 57.7 ± 1.7 ml·100 g^−1^·min^−1^ in YG, 48.6 ± 1.0 ml·100 g^−1^·min^−1^ in OLD, 44.7 ± 1.0 ml·100 g^−1^·min^−1^ in MCI, 40.8 ± 2.0 ml·100 g^−1^·min^−1^ in AD1, 34.5 ± 1.5 ml·100 g^−1^·min^−1^ in AD2, and 30.1 ± 2.5 ml·100 g^−1^·min^−1^ in AD3 and significant post-hoc comparisons across MCI and different AD stages (Figure 1C).

### ICA blood flow

**Figure 2:**
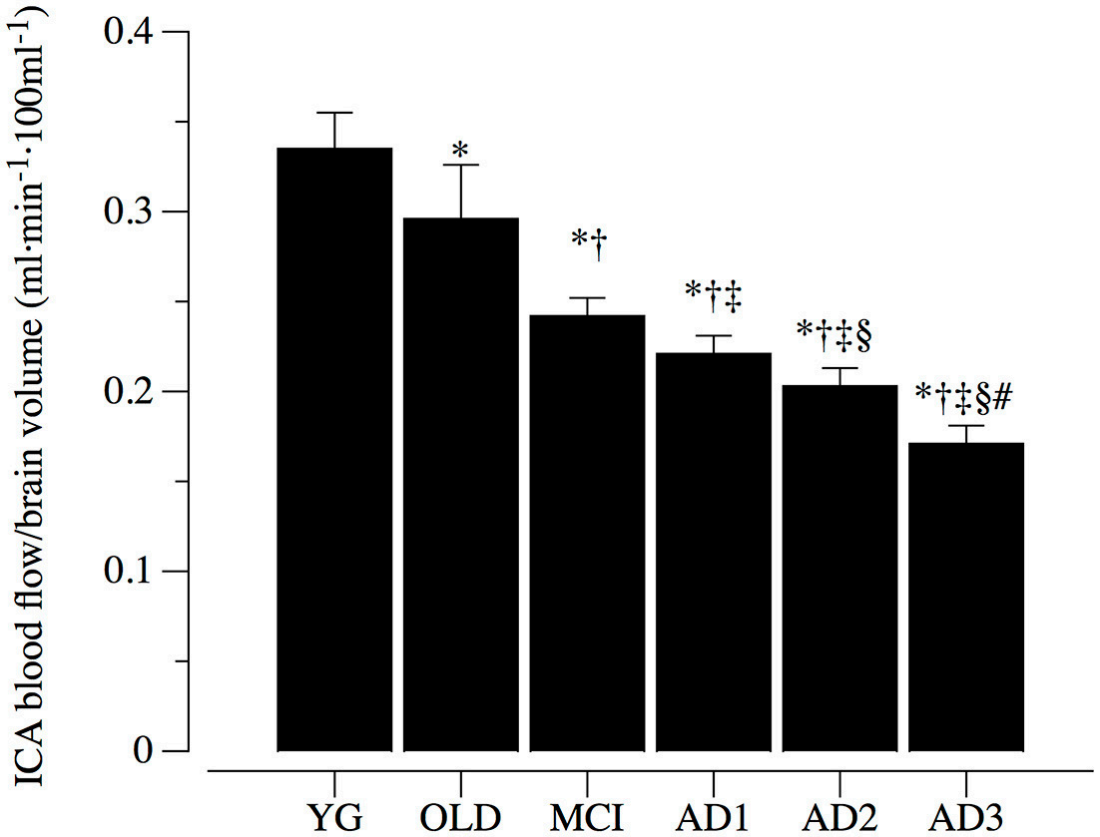
Internal carotid artery blood flow normalized to brain volume. Internal carotid artery (ICA) blood flow normalized to brain volume in healthy young (YG) and healthy old (OLD) controls, patients with mild cognitive impairment (MCI), and with different severity of AD, namely mild AD (AD1), moderate AD (AD2), and severe AD (AD3). Data are presented as mean ^±^S.E.; ^*^significantly different vs. YG group. ^†^Significantly different vs. OLD group. ^‡^Significantly different vs. MCI group. ^§^Significantly different vs. AD1 group. ^#^Significantly different vs. AD2 group.

Resting blood flow in the ICA normalized to total brain tissue volume, taken as marker of extracranial blood flow, is illustrated in Figure 2. Among the six groups there was a progressive significant reduction in ICA blood flow (*p* < 0.05). Specifically, blood flow in the ICA was 0.34 ± 0.02 ml·min^−1^·100 ml^−1^ in YG, 0.30 ± 0.03 ml·min^−1^·100 ml^−1^ in OLD, 0.24 ± 0.01 ml·min^−1^·100 ml^−1^ in MCI, 0.22 ± 0.01 ml·min^−1^·100 ml^−1^ in AD1, 0.20 ± 0.01 ml·min^−1^·100 ml^−1^ in AD2, and 0.17 ± 0.01 ml·min^−1^·100 ml^−1^ in AD3 subjects (Figure 2). Post-hoc comparisons were significant across MCI and different AD stages.

### FA blood flow

**Figure 3:**
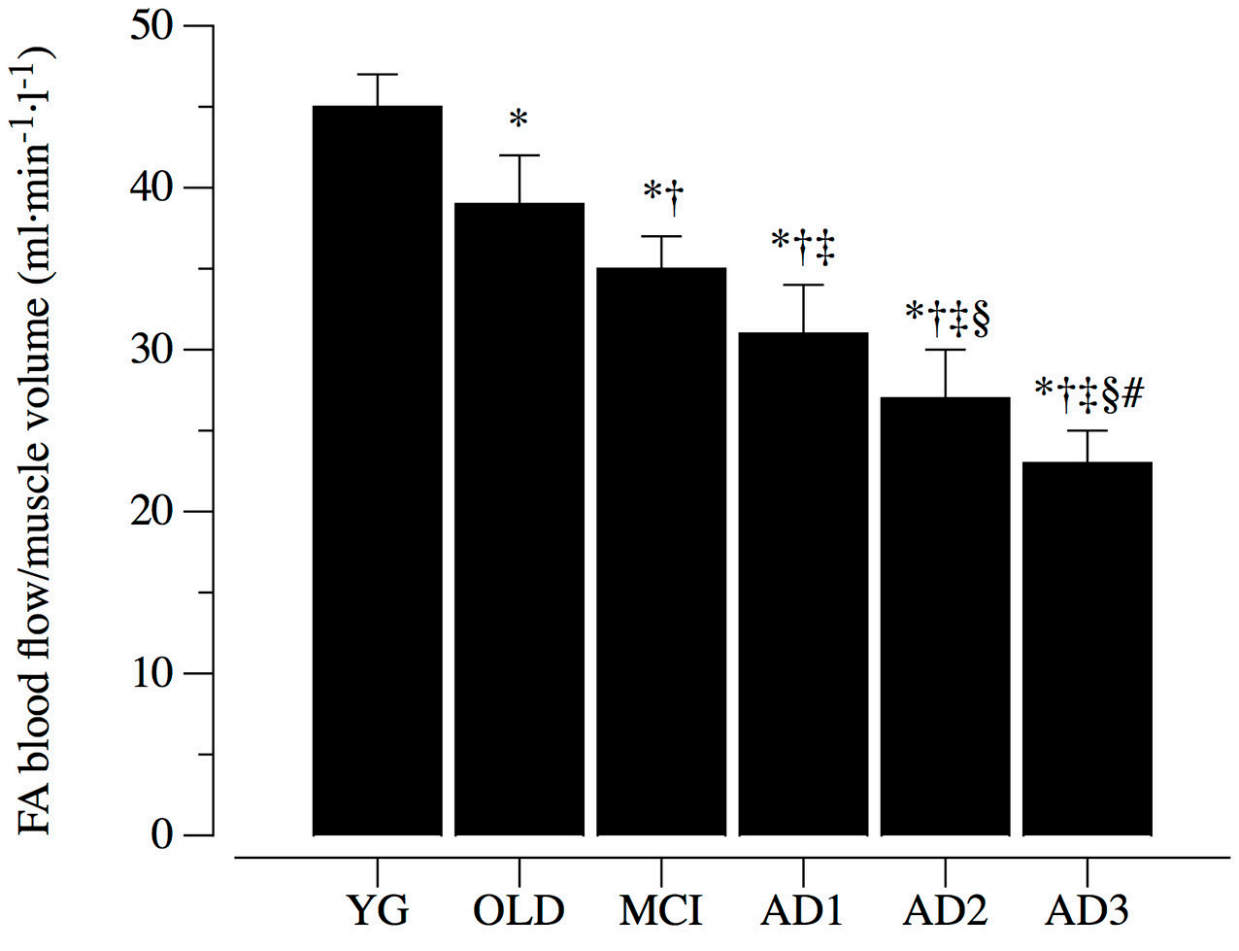
Femoral artery blood flow normalized to muscle limb volume. Femoral artery (FA) blood flow normalized for lower limb muscle volume in healthy young (YG) and healthy old (OLD) controls, patients with mild cognitive impairment (MCI), and with different severity of AD, namely mild AD (AD1), moderate AD (AD2), and severe AD (AD3). Data are presented as mean ^±^S.E.; ^*^Significantly different vs. YG group. ^†^Significantly different vs. OLD group. ^‡^Significantly different vs. MCI group. ^§^Significantly different vs. AD1 group. ^#^Significantly different vs. AD2 group.

Resting blood flow in the FA normalized to the lower limb muscle volume, is illustrated in Figure 3. With a similar trend of the ICA blood flow, the FA hemodynamic was significantly attenuated (*p* < 0.05) compared to the YG (45 ± 2 ml·min^−1^·l^−1^) in the OLD (39 ± 3 ml·min^−1^·l^−1^) and even more so in the MCI, AD1, AD2 and AD3 (35 ± 2 ml·min^−1^·l^−1^, 31 ± 3 ml·min^−1^·l^−1^, 27 ± 3 ml·min^−1^·l^−1^, and 23 ± 2 ml·min^−1^·l^−1^) respectively (Figure 3). Post-hoc comparisons were significant across MCI and different AD stages.

### NO bioavailability and systemic vascular function

**Figure 4:**
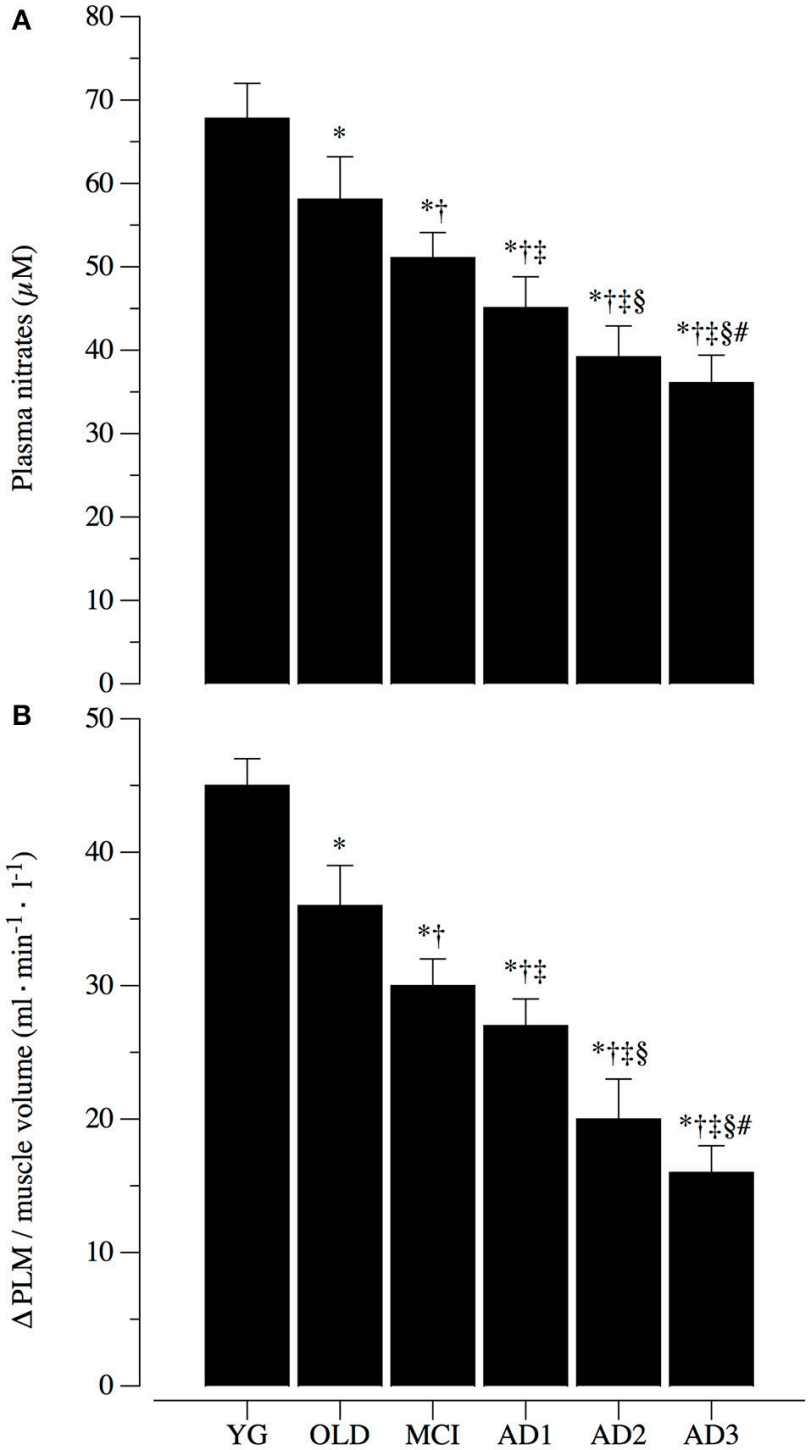
Nitric oxide bioavailability and systemic vascular function. Nitric oxide (NO) bioavailability was determined by plasma levels of nitrates **(A)** and passive limb movement induced hyperemia normalized for muscle volume (ΔPLM; **B**). Systemic vascular function determined via ΔPLM/muscle volume is represented in **(B)** in healthy young (YG) and healthy old (OLD) controls, patients with mild cognitive impairment (MCI), and with different severity of AD, namely mild AD (AD1), moderate AD (AD2), and severe AD (AD3). Data are presented as mean ^±^S.E.; ^*^Significantly different vs. YG group. ^†^Significantly different vs. OLD group. ^‡^Significantly different vs. MCI group. ^§^Significantly different vs. AD1 group. ^#^Significantly different vs. AD2 group.

Plasma levels of nitrates and PLM induced hyperemia, which were used as markers of NO bioavailability and systemic vascular function, are illustrated in Figure 4. Both these markers of NO bioavailability showed a clear and progressive reduction across the groups. Specifically, plasma level of nitrates was significantly reduced (*p* < 0.05) from YG to OLD, MCI and through AD stages, with values of 67.8 ± 4.2 μM in the YG, 58.1 ± 5.1 μM in OLD, 51.1 ± 3.0 μM in MCI, 45.1 ± 3.7 μM in AD1, 39.2 ± 3.7 μM in AD2, and 36.1± 23.3 μM in AD3 (Figure 4A). Similarly, ΔPLM hyperemia normalized for the muscle volume was significantly reduced (*p* < 0.05) among the 6 groups, with values of 45 ± 2 ml·min^−1^·l^−1^ in YG, 36 ± 3 ml·min^−1^·l^−1^ in OLD, 30 ± 2 ml·min^−1^·l^−1^ in MCI, 27 ± 2 ml·min^−1^·l^−1^ in AD1, 20 ± 3 ml·min^−1^·l^−1^ in AD2, and 16 ± 2 ml·min^−1^·l^−1^ in AD3 (Figure 4B). Post-hoc comparisons were significant across MCI and different AD stages for both measures.

### Correlation between NO bioavailability, systemic vascular function and cortical, extracranial, and peripheral blood flow

**Figure 5:**
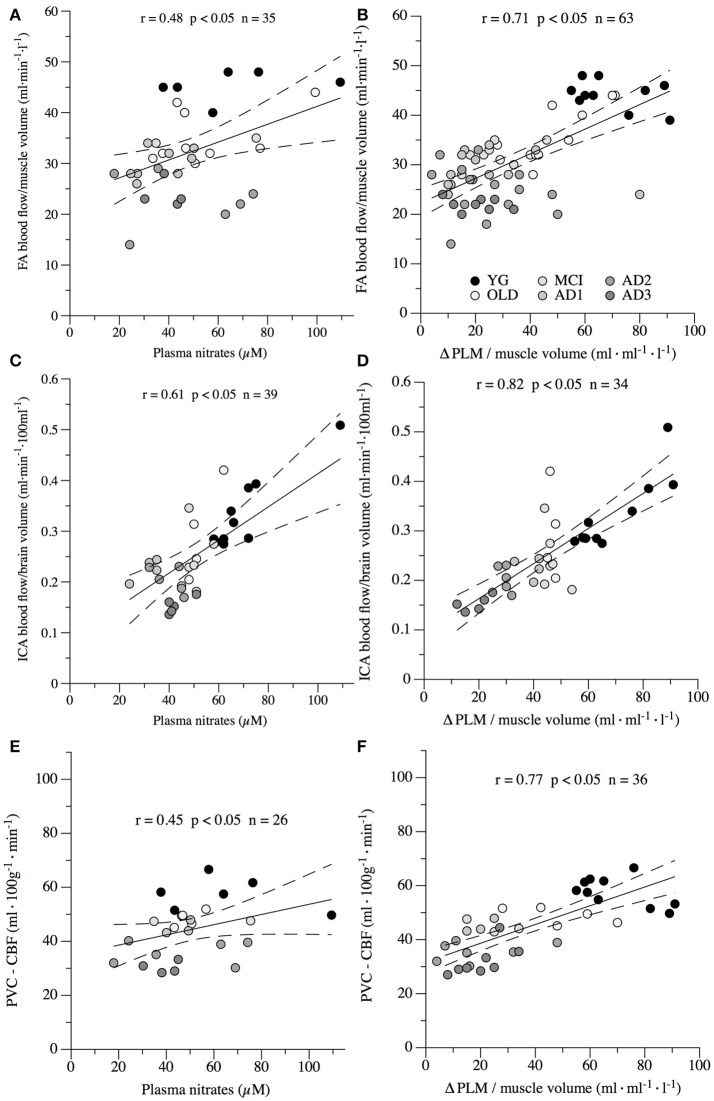
Correlations between nitric oxide bioavailability, systemic vascular function and cortical (PVC–CBF), internal carotid artery (ICA) and femoral artery (FA) blood flow normalized for brain and muscle volume, respectively. Nitric oxide (NO) bioavailability was determined by plasma levels of nitrates **(A,C,E)** and passive limb movement induced hyperemia normalized to muscle volume (ΔPLM; **B,D,F**). Systemic vascular function determined via ΔPLM/muscle volume is represented in **B,D,F** in healthy young (YG) and healthy old (OLD) controls, and patients with mild cognitive impairment (MCI), and with different severity of AD, namely mild AD (AD1), moderate AD (AD2), and severe AD (AD3). Each point represents a single subject, dashed lines represents interval of confidence.

Correlations between plasma levels of nitrates, PLM-induced hyperemia, which were used as markers of NO bioavailability and systemic vascular function, cortical perfusion, extracranial blood flow, and peripheral circulation are illustrated in Figure 5. Specifically, significant correlations were found between plasma levels of nitrates and FA blood flow normalized to the limb muscle volume (Figure 5A; *r* = 0.48, *p* < 0.05), ICA blood flow normalized for brain volume (Figure 5C; *r* = 0.61, *p* < 0.05), and PVC–CBF (Figure 5E; *r* = 0.45, *p* < 0.05). Interestingly, also values of ΔPLM/muscle volume were significantly correlated with FA blood flow/muscle volume (Figure 5B; *r* = 0.71, *p* < 0.05), ICA blood flow/brain volume (Figure 5D; *r* = 0.82, *p* < 0.05), and PVC–CBF (Figure 5F; *r* = 0.77, *p* < 0.05).

## Discussion

**Figure 6:**
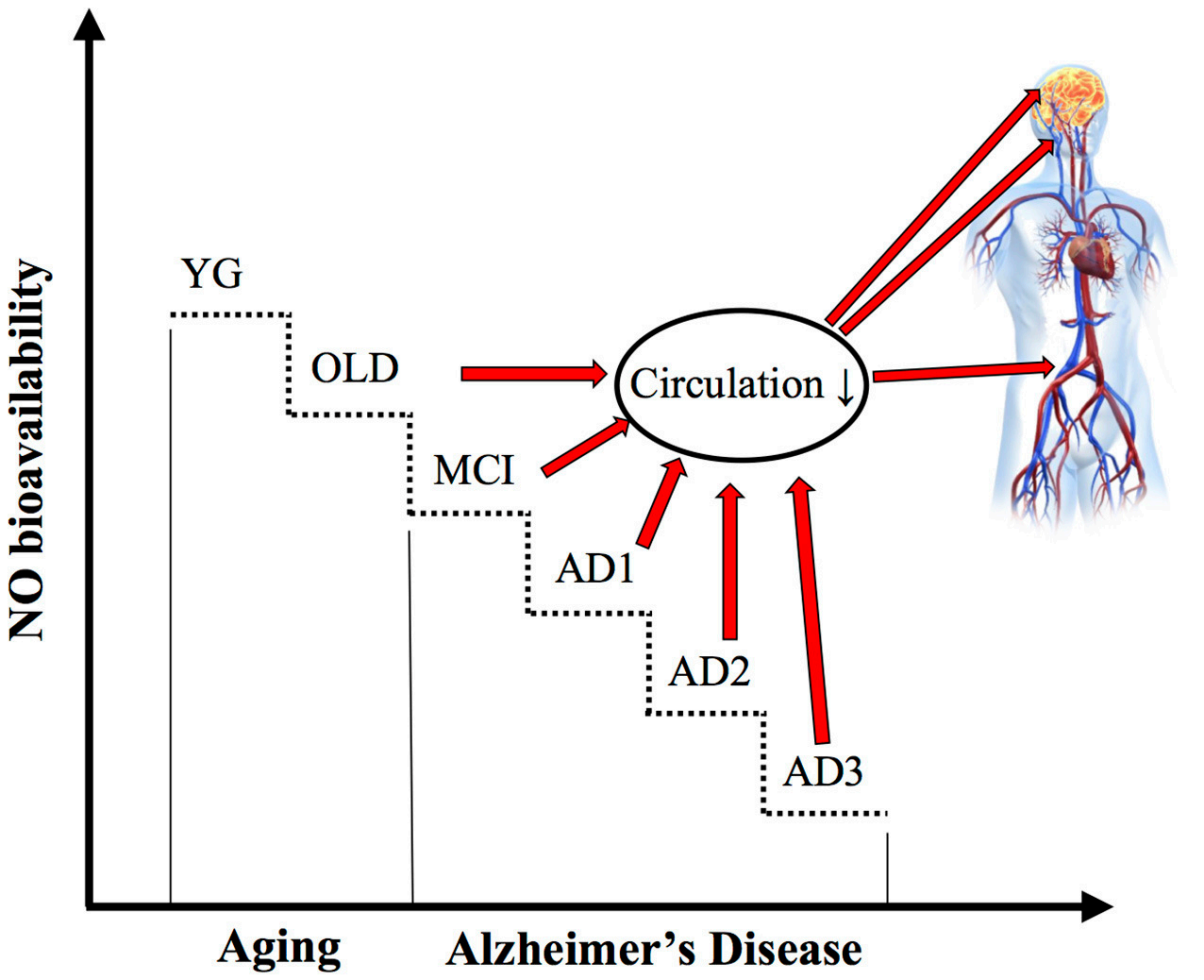
schematic illustration: Impact of nitric oxide bioavailability on the progressive cerebral and peripheral circulatory impairments during aging and Alzheimer's disease.

Although the association between reduction of NO bioavailability, cortical hypoperfusion and systemic vascular dysfunction has been already investigated in relation to AD onset in murine models, the mechanistic role of NO depletion in the reduction of extracranial blood flow and impairment of cortical and peripheral circulation in humans with AD has received so far only little attention. In the present study, we assessed ICA blood flow, cortical perfusion, and peripheral circulation in patients with MCI and different stages of AD and compared them to young and old healthy controls. Additionally, NO bioavailability was determined in the six groups of participants via plasma NO metabolites. A further indicator of endothelial NO bioavailability and systemic vascular function was estimated with PLM induced hyperemia. The main finding of this study was that ICA blood flow, cortical perfusion, peripheral circulation, and systemic vascular function were reduced in OLD vs. YG controls, and progressively further decreased in parallel to MCI and AD severity. These data suggest a pivotal role of AD, *per se*, to these vascular abnormalities. Though the causative relationship between NO bioavailability, central and peripheral circulation is still matter of debate, according to our hypothesis, circulation impairment was associated with NO depletion (Figures 5, 6).

### Evidence that AD, *per se*, affects NO bioavailability and blood flow

Indeed, the first risk factor for AD is advanced age (Reitz et al., 2011; Reitz and Mayeux, 2014). It should also be noted that, independent of AD, reduced availability of NO, which is dramatically decreased in the aged population, results in major detrimental alterations of vascular function, including vasoconstriction and hypertension, leading to atherosclerosis. On the other hand, recent literature highlighted that AD is highly correlated to systemic vascular dysfunction (Iturria-Medina et al., 2016). Interestingly, current literature suggests the key role of NO depletion in the pathogenesis of neurodegenerative disease (Katusic and Austin, 2014). Therefore, we may speculate that both age and AD appear to be involved in the decline of the systemic and cerebral vascular function likely because of endogenous NO reduced bioavailability. However, the contribution of AD, *per se*, to this putative pathophysiological mechanism is still unclear. To better answer this point, we explored NO bioavailability, and cortical, ICA and peripheral blood flow in a group of patients with similar age (~78 years) but different cognitive impairment (i.e., MCI and AD of increasing severity) and compared them to young and old (aged-matched) controls. This integrative and comprehensive approach to vascular changes in AD showed changes in NO bioavailability and cortical, extracranial, and peripheral circulation in patients with AD and suggested that they are directly associated with AD and not to aging (Figure 6).

### Evidence that cortical circulation is impaired in parallel to AD severity

Although the traditional “amyloid cascade hypothesis” proposed by Hardy and Higgins (Hardy and Higgins, 1992) indicates cortical deposition of Aβ fragments, neurofibrillary tangles and reactive microgliosis as the hallmark of AD, converging evidence underscores the importance of other pathogenetic mechanisms in AD, including oxidative stress, inflammation, and mitochondrial dysfunction (Swerdlow, 2011; Zenaro et al., 2016). In this multifaceted scenario, has germane the hypothesis that vascular dysfunction in the cortex plays a key role in AD pathophysiology (De La Torre, 2010). Specifically, impairment of cortical perfusion appears to be highly correlated to AD onset, implicating a pivotal role of vascular dysfunction and CAA. The recent development in MRI techniques led to ASL, a new advanced and non-invasive approach to brain perfusion measurement, coupled with more precise post-processing analysis. Our data are in agreement with and extend previous knowledge in this field (Detre et al., 1992), indicating that cortical hypoperfusion is not only associated with the presence of AD, but most important, is well correlated with the severity of dementia, NO bioavailability, and systemic vascular dysfunction (Figures 1, 5). The relevance of this result is particularly important because current biomarkers of AD, such as cerebrospinal fluid tau/Aβ fragments and positron emission tomography amyloid imaging, are known to change non-linearly throughout the progression of AD (Jack et al., 2013). We may speculate that cognitive impairment might be more related to cortical perfusion, while Aβ deposition takes place in the early/preclinical phases of AD. If confirmed in longitudinal studies, this hypothesis might indicate new therapeutic strategies for AD.

### Evidence that ICA blood flow is impaired in parallel to AD severity

Indeed, most of the literature related to circulatory dysfunction associated with AD was focused on the cortical areas, which are primarily affected in AD (Du et al., 2006). Our data (Figures 2, 3) are in agreement with recent reports of a coupling between cortical hypoperfusion and a reduction of blood flow from ICA (Maalikjy Akkawi et al., 2003; Liu et al., 2014; Clark et al., 2017), and highlight that AD-related circulation impairment is not confined to the cortex, but is more likely the effect of a systemic vascular dysfunction (De La Torre, 2009, 2010). Moreover, the recognition of ICA blood flow reduction in parallel to AD severity implies an exacerbated cortical hypo perfusion in this population. These findings suggest that cortical perfusion changes measured via ASL are strongly dependent on abnormal inflow from extracranial arteries (Clark et al., 2017).

### Evidence that peripheral vascular function is impaired in parallel to AD severity

Along with cortical alteration of blood flow, few studies reported evidence that peripheral vascular dysfunction determined by ankle-to-brachial index, flow-mediated dilation, intima-media thickness, and endothelial microvascular response to acetylcholine are associated with AD (Dede et al., 2007; Khalil et al., 2007; Laurin et al., 2007; Tachibana et al., 2016). This literature suggests that systemic vascular impairments are determined by AD, or from a different point of view, that systemic vascular dysfunctions may trigger AD (De La Torre, 2004). In this complex cause-effect scenario, the peripheral vascular difference between the vascular dementia and AD has been accounted, and AD appears, *per se*, to be associated with a significant reduction of systemic vascular function. Data from the current study (Figures 3–5) confirm this view and extend the relationship between AD onset and circulatory impairment up to the more advanced phases of the disease.

### Evidence that depletion in NO bioavailability is correlated with reduction of cortical, extracranial, and peripheral blood flow

Nitric oxide, an unstable free radical endogenously synthesized by several cell-types, exerts various biological regulatory functions at peripheral level in the nervous and cardiovascular systems (Loscalzo and Welch, 1995; Calabrese et al., 2007). Indeed, depletion of NO and endothelial nitric oxide synthase enzymatic activity, as a major endogenous source of NO, are one of the mechanisms in the pathogenesis of endothelial dysfunction in both cerebral and peripheral blood vessels (Katusic and Austin, 2014). Interestingly, recent literature has underlined the key role of NO depletion in the early stage of neurodegenerative disorders, as well as in their progression (Katusic and Austin, 2014). In a recent murine study, Merlini and coauthors (Merlini et al., 2017) revealed that reduced NO bioavailability mediates cerebro-arterial and peripheral dysfunction independently from CAA. Interestingly, and similarly to the data retrieved in our human model, endothelium-dependent vasorelaxation was significantly impaired in both basilar and femoral arteries of 15-month-old Swedish arctic (SweArc) transgenic AD mice compared with that of age-matched wild-type and 6-month-old SweArc. This vascular impairment was accompanied by significantly reduced levels of cyclic GMP, demonstrating the central role of NO bioavailability in the pathogenesis and development of AD. Due to the transitory and unstable nature of this free radical, several studies have determined the bioavailability of NO via plasma levels of nitrite and nitrate (Casey et al., 2007, 2010). Interestingly, this literature indicates a strong positive relationship between plasma level of nitrite and nitrate and systemic vascular function (Casey et al., 2007, 2010). The present data are in agreement with the above-mentioned animal and human studies, and support the hypothesis that, in humans, the depletion in NO bioavailability is correlated with reduction of cortical, extracranial, and peripheral blood flow during aging and in parallel to AD severity (Figures 4–6).

### Other physiological considerations

The recent literature underlined that augmenting physical activity and fitness can protect NO bioavailability, attenuating the deleterious effects of advancing age on vascular function (Groot et al., 2016). Therefore, particular attention on the determination of the physical activity level is needed in order to better describe the net effect of aging and AD to the systemic vascular function. As expected, our results indicate that in comparison to the YG, healthy elderly and patients with AD, were more sedentary (Table 1). These data suggest that the reduction of systemic vascular function and NO bioavailability of these groups are likely affected by their low-level of physical activity. However, it is important to note that the IPAQ values among OLD, MCI, AD1, AD2, and AD3 were similar, implicating that in these age-matched groups, aging and level of physical activity are not responsible of the progressive reduction of NO bioavailability and vascular dysfunction. Another physiological factor important to mention in relation to the cerebral blood flow assessment is the level of CO_2_. In fact, due to its vasodilatory effect on the conduit intra- and extracranial arteries, hypercapnia is routinely utilized for the evaluation of maximal cerebral perfusion. Therefore, the determination of ExpCO_2_ is required in order to normalize the cerebral blood flow. The data of resting ExpCO_2_ (Table 1) were similar in the 6 groups, implicating that ExpCO_2_ did not play a role in the changes of cerebral blood flow in our subjects. Indeed, resting blood flow to a specific organ is affected by its volume of metabolically active tissue. As previously described in the text, partial volume-corrected cortical and WM flow maps were created for each subject, and FA and ICA blood flow were normalized to leg muscle volume (thigh + lower leg volume) and total brain tissue volume (cortical, subcortical gray matter and white matter volumes including the brainstem and cerebellum), respectively (Liu et al., 2014; Venturelli et al., 2014). Indeed, basal metabolism is another important physiological factor affected by aging (Venturelli et al., 2013) and AD (Venturelli et al., 2016), that may contribute to the resting blood flow changes during aging and AD. Interestingly, our data of resting oxygen uptake (Table 1) indicate similar basal metabolism in the six groups of subjects, suggesting that this physiological factor is not playing a direct role in the progressive changes of brain and skeletal muscle blood flow. It is important to mention that NO is a free radical playing several positive regulatory functions at cellular and systemic level. However, it is well established that elevated levels of free radicals have a plethora of deleterious effects on the vascular and nervous system during aging and AD, primarily associated with mitochondrial dysfunction. Indeed, Sewrdlow and Khan (Swerdlow, 2011) hypothesized the “*mitochondrial cascade hypothesis*” in AD, whereby mitochondrial dysfunction accumulates over the disease course, resulting in both symptoms and neuropathological aspects of AD (Swerdlow et al., 2010). It is believed that mitochondrial dysfunction precedes Aβ formation, increasing reactive oxygen species (ROS) and oxidative stress, which, in turn, may facilitate overproduction of Aβ (Morris et al., 2014). In AD, mitochondrial damage is characterized by decreased respiratory chain complexes activities, where complexes III and IV are typically involved, causing ROS overproduction and reduced ATP synthesis (Marques-Aleixo et al., 2012; Cadonic et al., 2016; Pedrinolla et al., 2017). In this regard, brain tissues are metabolically very active and are particularly susceptible to the damaging effects by ROS. In case of AD, ROS have been reported within those brain regions, such as the cerebral cortex and hippocampus, which undergo selective neurodegeneration (Bhat et al., 2015). Interestingly, a large body of evidence shows that AD patients have oxidative metabolism dysfunction in both the central nervous system (CNS) and peripheral tissues (i.e., vascular endothelial cells, platelets) suggesting that pathological changes co-exist in brain and non-neural tissues (Morris et al., 2014; Cadonic et al., 2016). Moreover, recent studies suggest that mitochondria ROS overproduction contribute to accelerate the development of the senescent phenotype in endothelial cells, impairing regenerative and angiogenic capacity of the endothelium, promoting atherosclerosis by altering the secretion of cytokines, growth factors, and protease in the vascular wall (Dai et al., 2012; El Assar et al., 2013).

Other potential confounding factors that may have influenced, at least in part, our findings include the deconditioning due to AD, the age-related aortic stiffness and progressive impairment in diastolic heart functions (Pase et al., 2016).

## Author contributions

MV performed the experiments, analyzed the data, prepared the figures, and drafted the manuscript. APe performed the experiments, analyzed the data, and drafted the manuscript. IB performed the experiments, analyzed the data, and drafted the manuscript. CF performed the experiments, analyzed the data, and drafted the manuscript. NS interpreted the results of experiments, and drafted the manuscript. ST performed the experiments, interpreted the results of experiments, and drafted the manuscript. EM performed the experiments, interpreted the results of experiments, and drafted the manuscript. LC performed the experiments, interpreted the results of experiments, and drafted the manuscript. AS performed the experiments, interpreted the results of experiments, and drafted the manuscript. APi performed the experiments, interpreted the results of experiments, and drafted the manuscript. MR interpreted the results of experiments, and drafted the manuscript. FP interpreted the results of experiments, and drafted the manuscript. FS edited, revised, and approved the final version of manuscript.

### Conflict of interest statement

The authors declare that the research was conducted in the absence of any commercial or financial relationships that could be construed as a potential conflict of interest.
